# Ca_v_1.2 Activity and Downstream Signaling Pathways in the Hippocampus of An Animal Model of Depression

**DOI:** 10.3390/cells9122609

**Published:** 2020-12-04

**Authors:** Cristian Moreno, Tamara Hermosilla, Paulina Hardy, Víctor Aballai, Patricio Rojas, Diego Varela

**Affiliations:** 1 Program of Physiology and Biophysics, Institute of Biomedical Sciences (ICBM), Faculty of Medicine, Universidad de Chile, Santiago 8380453, Chile; cristian.moreno.n@usach.cl (C.M.); thermosillabellenger@gmail.com (T.H.); Aballai.victor@gmail.com (V.A.); 2 Laboratory of Neuroscience, Faculty of Chemistry and Biology, University of Santiago de Chile, Santiago 8320000, Chile; p.hardy.t@gmail.com (P.H.); patricio.rojas.m@usach.cl (P.R.); 3 Millennium Nucleus of Ion Channels-Associated Diseases (MiNICAD), Universidad de Chile, Santiago 8380453, Chile

**Keywords:** l-type calcium current, major depression disorder, hippocampus

## Abstract

Functional and morphological modifications in the brain caused by major mood disorders involve many brain areas, including the hippocampus, leading to cognitive and mood alterations. Ca_v_1.2 channel expression has been found to increase in animals with depressive-like behaviors. Calcium influx through these channels is associated with changes in excitation-transcriptional coupling by several intracellular signal pathways that are regulated by its C-terminus region. However, which of these signaling pathways is activated during the development of depressive-like behaviors is not known. Here, we evaluate the phosphorylation and expression levels of crucial kinases and transcription factors at the hippocampus of rats after 21 days of chronic restraint stress. Our results show that rats subjected to CRS protocol achieve less body weight, have heavier adrenal glands, and exhibit depression-like behaviors such as anhedonia, behavioral despair and decreased social interaction. Ca_v_1.2 mRNA and protein expression levels, plus l-type calcium current amplitude, are also increased in treated rats when compared with control animals. Out of the three main signaling pathways activated by l-type currents, we only observed an increment of CaM-NFAT axis activity with the concomitant increment in Fas ligand expression. Thus, our results suggest that CRS activates specific pathways, and the increased expression of Ca_v_1.2 could lead to neuronal death in the hippocampus.

## 1. Introduction

Major depression disorder is the most common psychiatric disorder in the human population [1]. Although biological studies have provided progress in understanding the development of depression, a considerable number of patients do not show improvements with the current pharmacological treatments [2,3]. Historically, depression has been correlated with monoamine bioavailability alterations at the synaptic level [4]; however, some studies have associated the development of depression with ion channel alterations, including AMPA and NMDA type glutamate receptor, GABA-A receptor, potassium channels, and calcium channels [5,6,7,8].

Genome-wide association studies (GWAS) exhibit a strong link between Rs1006737 polymorphisms in the CACNA1C gene (that encodes for the Ca_v_1.2 calcium channel) and the development of mood disorders such as bipolar disorder and major depression [9,10,11]. These polymorphisms are associated with increased Ca_v_1.2 mRNA and increased l-type calcium currents in induced neurons obtained from skin fibroblasts of individuals harboring the rs1006737 haplotype, which has been associated with depressive-like behaviors [12]. Ca_v_1.2 protein expression is also increased in animals subjected to unpredictable stress [13,14,15]: these animals also developed depressive-like behaviors which can be prevented in Ca_v_1.2^+/−^ animals [14].

Somatic calcium influx in hippocampal neurons is mainly associated with the NMDA glutamate receptor and the l-type calcium channels (LTCC) [16,17]. LTCCs in neurons are located at the somatodendritic level and comprise the Ca_V_1.3, and the Ca_v_1.2 isoforms, the latter being the isoform with higher expression levels in hippocampal neurons [18]. This channel plays a fundamental role in transforming electrical stimuli to a chemical signal and is crucial in the excitation–transcription coupling and apoptosis [19,20].

The C-terminal domain (C_term_D) of Ca_v_1.2 harbors a series of aminoacidic motifs capable of regulating gene transcription by interacting with different proteins, like calmodulin (CaM), which is necessary for CREB activation [19,21]. Calcium influx through Ca_v_1.2 channels induces NFAT nuclear translocation via calcineurin activation [22,23,24]. This transcription factor is related to ERK activation, which increases specific gene expression and apoptosis through the Fas ligand/Fas death pathway [25,26]. 

The C-terminus of Ca_v_1.2 encodes a transcription factor, known as calcium-channel-associated transcriptional regulator (CCAT) [27], and although it is unclear if its activity depends on proteolytic cleavage of the Ca_v_1.2 protein or an independent internal promoter in the CACNA1C gene [28], it is accepted that CCAT regulates neurite extension in neurons and dental pulp, and inhibits Ca_v_1.2 expression in cardiomyocytes [29,30]. However, which of these pathways correlates with increased l-type calcium current during the development of depressive-like behaviors has not been explored. 

We studied the effect of chronic stress on the activity of different pathways associated with Ca_v_1.2 activation in hippocampal neurons. For this, we used an animal model of depression involving three weeks of chronic restraint stress (CRS) in juvenile rats. Our results showed that animals subjected to CRS gain less body weight, have larger adrenal glands, and show behavioral modifications assessed by the forced swimming test (FST), and a sucrose preference test (SPT). Social interaction tests (SIT) indicated that animals subjected to CRS also displayed decreased social interaction. In addition, pyramidal neurons of the hippocampus of CRS-subjected rats presented an increase in Ca_v_1.2 mRNA levels, protein expression, and l-type calcium current amplitude, compared with control animals. Of the signaling pathways that are activated by l-type calcium current, no changes in CCAT, ERK or CaMKII, and CaMKIV activity were found in animals subjected to CRS; in contrast, we observed increased CaM-NFAT axis activity with the concomitant increment in Fas ligand (FasL) expression, but not with other genes that are known to be regulated by NFAT. In conclusion, our results suggest that, in the hippocampus, chronic restraint stress activates specific pathways, and the increased expression of Ca_v_1.2 in the hippocampus could lead to neuronal death.

## 2. Materials and Methods

### 2.1. Animals

Male Sprague-Dawley rats were bred under stable conditions of temperature (22 ± 2 °C) and humidity (50 ± 5%), with food and water ad libitum under a 12-h light–dark cycle in the Animal Facility from the Facultad de Química y Biología de la Universidad de Santiago de Chile (Santiago, Chile); the illumination was 300 ± 20 lx. Control animals were kept in a separate room to the CRS-subjected group of animals. All studies were performed in accordance with the guidelines and approval of Universidad de Santiago de Chile and Universidad de Chile Institutional Bioethical Committee. 

### 2.2. Chronic Stress

Male Sprague-Dawley rats (125–150 gr, P42), were grouped into control and experimental groups. The experimental group was subjected to chronic restraint stress (CRS) for 21 consecutive days by placing the animals daily in well ventilated and transparent acrylic restrainers (6 × 6 × 18 cm) for two hours (9–11 am), within their home cages. Rats were weighed individually, every day at 8:30 a.m., starting two days before initiation of the restraint stress protocol, and finishing two days after the protocol. Weight gains were established as the difference between the first and the last day. 

### 2.3. Forced Swimming Test

Forced swimming test was performed in a transparent water cylinder (40 cm in height and 20 cm in diameter, containing a 30-cm column of water at a temperature between 23–25 °C) [31]; freshwater was used for each rat. The test was done over two sessions: first, each animal was placed in the water for 15 min to allow habituation; the next day, animals were placed in the water for 5 min, and their behavior was recorded for offline analysis. Climbing, swimming, diving, and immobility (floating passively to keep their nose above water) times were evaluated by using AnyMaze software (Wood Dale, IL, USA). First latency was considered as the time that the rat took to become completely immobile for the first time in the test.

### 2.4. Sucrose Preference Test

The sucrose preference test was performed every seven days by placing each rat in a single cage with two bottles: one containing water and the other a 1% sucrose solution. The bottle’s position was changed every 12 h. Sucrose preference was calculated as the ratio between 1% sucrose solution intake over the total amount of water consumed for 24 h.

### 2.5. Three-Chamber Social Interaction Test

The social interaction test was evaluated in an interaction arena (40 cm height, 40 cm width, and 120 cm large) divided into three chambers. The animal is placed in the middle chamber and allowed to explore all three compartments, one with an animal inside a pencil cup and the other with an empty pencil cup. The social interaction test was subdivided into four steps, each one lasting 10 min and each separated by 10 min: (1) First exploration, (2) Interaction with a familiar subject, (3) Second arena exploration, and (4) Interaction with an unfamiliar subject. The analysis was performed offline with AnyMaze software (Wood Dale, IL, USA)

### 2.6. Sample Harvesting

After finishing the CRS protocol and the behavior assessment, rats were anesthetized with 2% saturated isoflurane and decapitated to obtain adrenal and whole hippocampus tissue. Both adrenal glands were weighed to compare controls and stressed animals. 

### 2.7. RT-PCR

Total RNA was obtained from whole hippocampal samples of control and stressed animals and was extracted using TRIzol^®^ reagent (Life Technologies, Inc., Grand Island, NY, USA) according to the manufacturer’s instructions. Samples were treated with DNAsaH l (Thermo Fisher Scientific, Waltham, MA, USA) before complementary DNA (cDNA) synthesis to eliminate genomic DNA. Single-stranded cDNA was reverse-transcribed from 100 ng total RNA by RT-PCR using the QPCR cDNA Synthesis Kit AffinityScript TM II (Stratagene Inc., La Jolla, CA, USA).

### 2.8. Semi-Quantitative PCR

Real-time semi-quantitative PCR assays were performed using a Stratagene MX300P thermal cycler (Stratagene, La Jolla, CA, USA). PCR amplification of the 18S RNA was used as an internal control. PCR reactions were done with Brilliant SYBR Green (Agilent technologies, Santa Clara, CA, USA) according to the manufacturer’s instructions. In the preliminary studies, PCR products were subjected to agarose gel electrophoresis and melting curves to confirm amplification specificity. After setting the PCR protocol and confirming the identity of the products, all subsequent PCR products were tested by melting curves. Results were analyzed according to the standard curve (correlation coefficient ≥0.98) [32]. Specific mRNA abundance was calculated as the ratio of the specific amount relative to the amount of 18S within each sample, determined in duplicate [33]. The primers used are listed in Table 1.

### 2.9. Western Blot

The whole hippocampus was lysed in cold RIPA buffer (50mM TRIS, 1% IGEPAL, 50 mM EDTA, 150 mM NaCl, 0.05% SDS, 1% Triton X-100, pH 7.4), supplemented with proteases and phosphatases inhibitors (Thermo Fisher Scientific, Waltham, MA, USA). Samples were homogenized with a UP50H ultrasonic processor (Hielscher Ultrasonics GmbH, Teltow, Germany) and lysates centrifuged at 13.000 rpm for 10 min. Proteins were separated by electrophoresis with 8% SDS-PAGE gels for 3 h at 90V and subsequently transferred to a nitrocellulose membrane. Proteins were visualized by ECL (Thermo Scientific) and pictures were obtained by ChemiScope 3200 mini (Clinx Science, Shanghai, China). The antibodies used are listed in Table 2.

### 2.10. Hippocampal Slices

Rats were transcardially perfused, under isoflurane anesthesia, with a cold dissection buffer (4 °C) containing (in mM): 92 NMDG, 2.5 KCl, 1.25 NaH_2_PO_4_, 30 NaHCO_3_, 20 HEPES, 25 glucose, 2 thiourea, 5 Na-ascorbate, 3 Na-pyruvate, 0.5 CaCl_2_·4H_2_O and 10 MgSO_4_·7H_2_O, pH titrated to 7.3–7.4 with concentrated hydrochloric acid [34]. Animals were decapitated and the brains were dissected and cut in transversal slices of 300 µm with a VT 1200 vibratome (Leica, Wetzlar, Germany) in cold dissection buffer supplemented with 2mM kynurenic acid (Sigma-Aldrich, San Luis, MO, USA). Slices were transferred to a storage chamber, kept at 32 °C, in Artificial Cerebrospinal Fluid (ACSF) containing (in mM): 124 NaCl, 2.8 KCl, 1.25 NaH_2_PO_4_, 26 NaHCO_3_, 10 Glucose, 0.4 ascorbic acid, 2 CaCl_2_, 2 MgCl_2_, pH 7.4.

### 2.11. Electrophysiology

Whole-cell slice recordings were obtained in CA1 pyramidal neurons. Borosilicate glass patch pipettes were pulled to 2–5 MΩ resistance and filled with an internal solution containing (mM): 100 CsCl, 0.5 CaCl_2_, 2MgATP, 2 NaGTP, 10 HEPES, 10 EGTA, 20 creatinine phosphate, 20 TEACl (300 mOsm/Kg and pH 7.4 adjusted with KOH). Hippocampal slices were infused with ACSF supplemented with 0.5mM Tetrodotoxin (TTX) (Cayman Chemical, Ann Arbor, MI, USA), 10mM TEA-Cl (Sigma-Aldrich), 5mM 4-AP (Sigma-Aldrich) and 5mM CsCl. Whole-cell recordings were obtained from the somas of CA1 pyramidal neurons visually identified under infrared differential interference contrast microscopy on a Nikon eFN600 microscope. Calcium currents were obtained at room temperature, using an Axopatch 1C amplifier and WinWCP acquisition software, low-pass-filtered at 5 kHz, and digitalized at 10 kHz. Series resistance was compensated to 80%. Cells with an access resistance bigger than 25 MΩ or an access resistance that increased more than 25% during the experiment were rejected. Five minutes after the whole-cell configuration was established, neurons were maintained at holding potential (−65 mV), and depolarizing pulses (200 ms duration) varying between −80 and 0 mV were delivered. Data analysis, currents fitting, and offline leak subtraction were performed in Clampfit 10 (Axon Instruments), and all curves were constructed with SigmaPlot 11 (Jandel Scientific) [35]. Current-Voltage (I-V) plots were fitted using a modified Botzmann equation (1)

(1) $$I=\frac{g_{max}*\left(V-E_{rev}\right)}{1+e^{-\left(V-V_{a}\right)/k}}$$

where *E_rev_* is the reversal potential, *g_max_* is the maximum slope conductance, *k* is the slope factor, and *V_a_* the half-activation voltage.

### 2.12. Statistical Analysis

All values are reported as mean ± sem (*n*). Statistical analysis of the data was performed with SigmaPlot 11 (Systat Software Inc., Chicago, IL, USA) using the unpaired Student’s *t*-test when the two groups were compared or one-way ANOVA if more than two groups were compared; in both cases, the significance was considered at *p* < 0.05 with a 95% power, except for Figure S1, where the power was 80%.

The total number of animals used in this study was 54 (24 for the control group and 30 for the CRS group); the detailed number for each experiment is summarized in Table 3.

## 3. Results

### 3.1. Behavioral Changes after CRS

**Figure 1:**
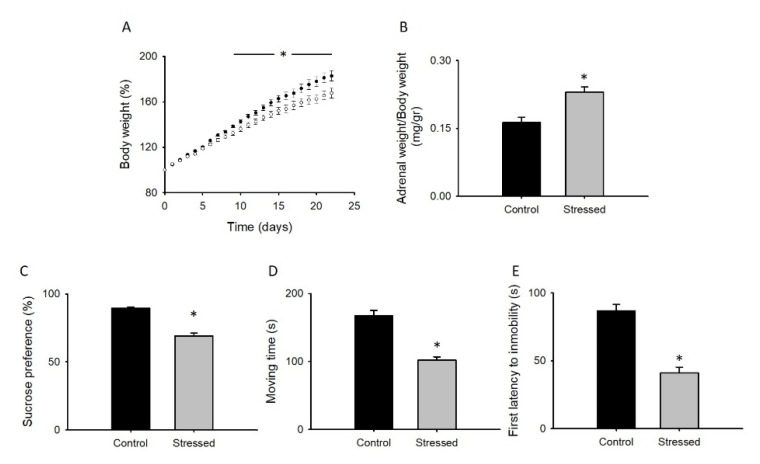
Rats subjected to CRS display behavioral modifications. (**A**) Body weight gain recorded daily of control animals (black circles) or rats during three weeks of CRS protocol. Bar graphs of adrenal glands weight normalized by total body weight (**B**), sucrose preference shown as percentage of sweetened water consumed with respect to the total consumed water (**C**). D and E shows the total time of swimming and climbing (**D**), and the latency to the first period of immobility (**E**) of animals during the forced swimming test. Bar graphs are mean ± SEM; black bars are control animals, and gray bars correspond to CRS-subjected animals. *n* > 9 * *p* value < 0.05.

As previously shown [36], animals subjected to CRS present significant reductions in body weight (BW) gain and an increased ratio of adrenal gland weight regarding whole BW when compared to control animals (Figure 1A,B). These differences are accompanied by anhedonic-like behavior reflected by a reduced sucrose preference (Figure 1C), a reduced first latency to immobility, and a lower total moving time (climbing, diving, and swimming) in the forced swimming test (FST), indicating a learned helplessness behavior in rats subjected to CRS (Figure 1D,E). As the FST measures coping strategy to an acute inescapable stress but does not predict depressive-like behavior by itself [37], we next sought to determine changes in sociability behavior.

**Figure 2:**
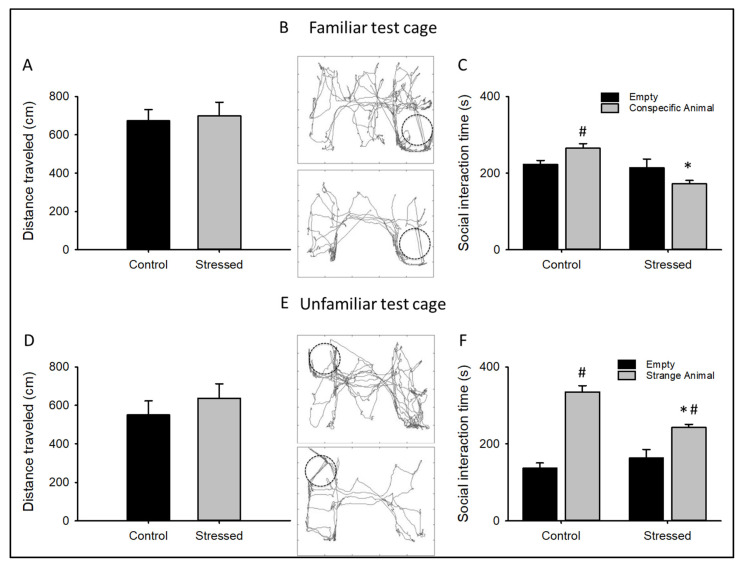
Chronic restraint stress affects the social interaction in rats: Graph plots showing the total distance traveled during the first exploration (**A**) or the time that each animal spent interacting with a familiar animal (**C**), the representative trajectory path of a control animal (up) or after three weeks of CRS protocol (down) in the interaction arena with a familiar animal is shown in (**B**). Graph plots showing the total distance traveled during the second exploration (**D**) or the time that each animal spent interacting with an unfamiliar animal (**F**), the representative trajectory path of a control animal (up) or after three weeks of CRS protocol (down) in the interaction arena with an unfamiliar animal is shown in (**E**). Bar graphs are mean ± SEM. *n* = 15 * *p* value < 0.05 between CRS-subjected animals and control; # *p* value < 0.05 when compared with the empty compartment.

To evaluate changes in sociability behavior, a three-chamber social interaction test was conducted with animals subjected to CRS and controls. The animal explores two separate compartments, one with a familiar and the other with an unfamiliar animal, measuring the time that was spent in each compartment, and comparing them with the time spent when the compartment was empty. Both animal groups spent more time in the proximity of an unfamiliar animal than in the empty compartment, suggesting a preference for social novelty (Figure 2F). Still, only the control group spent more in the proximity of a conspecific animal (Figure 2C), indicating less social affiliation in animals subjected to CRS. Moreover, the stressed group spent less time in the compartments with a familiar or unfamiliar animal when compared with the control group (Figure 2C,F). Altogether, these results suggest that the CRS protocol induces social-isolation-like behaviors.

No changes were observed in the total distance traveled in either explorations in the control group or in the stressed group, indicating no changes in motor activity (Figure 2A,D).

These results show that the CRS protocol induces learned helplessness, anhedonic-like, and social isolation-like behaviors, indicating that this stressed group of animals developed a depressive-like behavior.

### 3.2. Changes in Ca_v_1.2 after CRS

**Figure 3:**
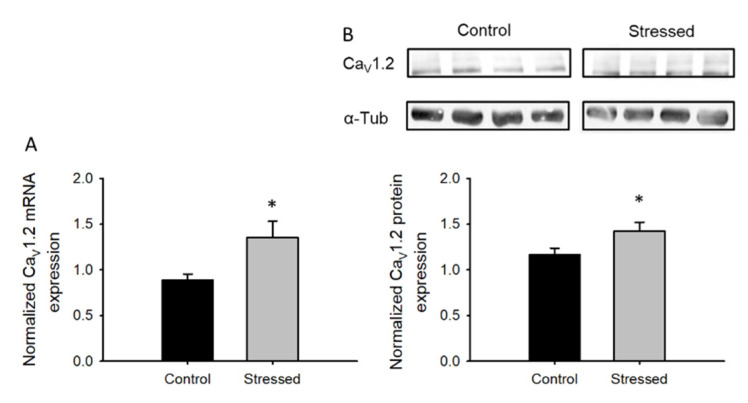
Chronic stress promotes the expression of Ca_v_1.2 subunit in whole hippocampal samples: (**A**) Bar graph of semi-quantitative PCR expression for the Ca_v_1.2 mRNA, normalized to 18S mRNA, isolated from whole hippocampus of juvenile rat controls (*n* = 8) or subjected to CRS for 3 weeks (*n* = 8). (**B**) Bar graph of Ca_v_1.2 protein expression from whole hippocampus lysates normalized to α-tubulin (*n* = 8), in the upper part, a representative immunoblots showing Ca_v_1.2 and α-tubulin protein expression is shown. Bar graphs are mean ± SEM; black bars are control animals, and gray bars correspond to CRS-subjected animals * *p* value < 0.05.

To evaluate if the CRS protocol induces changes at the molecular level, we measured the amount of Ca_v_1.2 mRNA transcripts from hippocampus samples. Hippocampus samples from animals subjected to CRS had increased Ca_v_1.2 mRNA levels (Figure 3A): this was accompanied by increased Ca_v_1.2 protein levels (Figure 3B).

**Figure 4:**
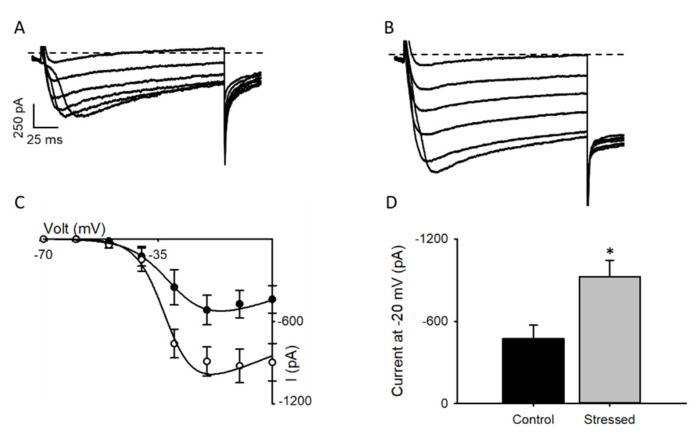
Chronic stress increases l-type calcium currents at CA1 pyramidal neurons: Representative whole-cell endogenous l-type Ca^2+^ current traces from pyramidal neurons of CA1 of hippocampal slices from control (**A**) or subjected to CRS protocol (**B**) animals. Currents elicited by a voltage step protocol from −70 to 0 in 10 mV increments, V_h_ = −80 mV. (**C**) Summary peak current I/V plots (mean ± sem) obtained from currents family, as shown in (A, black circles) and (B, white circles), where the black line represents the best fit to a Boltzmann equation. (**D**) Bar graph (mean ± sem) of the mean l-type current obtained at −20 mV (*n* = 7); black bars represent control animals, and gray bars CRS-subjected animals, * *p* value < 0.05.

The above results implicate that the l-type current in neurons from the hippocampus of animals subjected to CRS should be increased, a conclusion that was tested by recording this type of current in brain slices. Coherent with the data showing increased Ca_v_1.2 levels, stressed animals display an increase in mean calcium current density at all voltages (Figure 4A–C), with a relative I_max_ increase at −20 mV of almost 150% (Figure 4D). 

### 3.3. Changes in CCAT Activity after CRS

**Figure 5:**
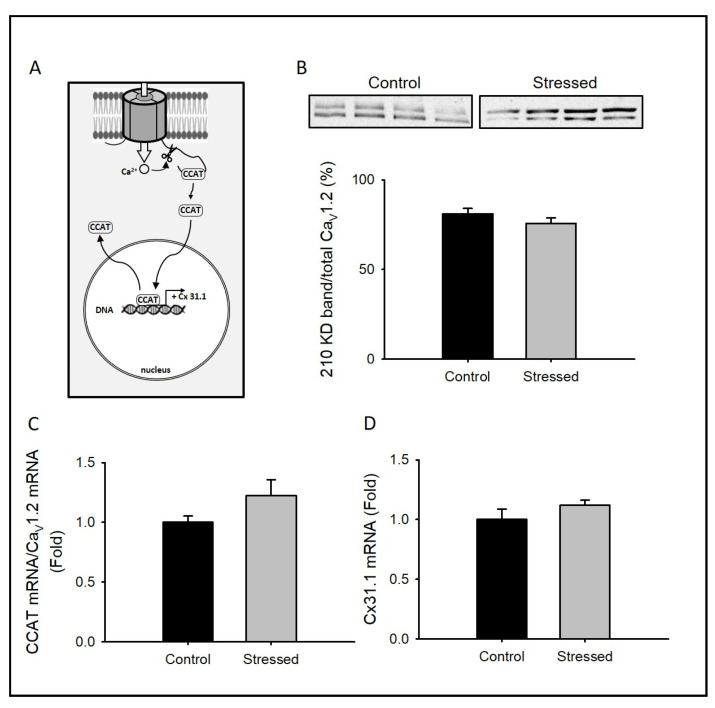
Calcium channel associated transcriptional regulator (CCAT)-dependent signaling in CRS-subjected animals: (**A**) Schematic diagram depicting the signaling pathway studied in this figure (**B**) Bar graph of 210 kD Ca_v_1.2 protein expression normalized to total Ca_v_1.2 protein expression (210 kD + 240 kD) from whole hippocampus lysates (*n* = 8), in the upper part, a representative immunoblot showing Ca_v_1.2 protein expression is shown. Bar graph of semi-quantitative PCR expression for the CCAT mRNA normalized to the Ca_v_1.2 mRNA (**C**) or Cx31.1 mRNA (**D**) isolated from whole hippocampus of juvenile rat controls (*n* = 8) or subjected to CRS for 3 weeks (*n* = 8). Bar graphs are mean ± SEM; black bars are control animals, and gray bars correspond to CRS-subjected animals.

One unique feature of the Ca_v_1.2 channel is that its C-terminus encodes an independent transcription factor that regulates the expression of a wide variety of neuronal genes, including some involved in neural differentiation [27]. The nuclear translocation of CCAT requires either C-terminal cleavage by calpain [27] or the activation of a promoter in exon 46 of CACNA1C that leads to a different transcriptional starting site (Figure 5A) [28].

To explore probable changes in the number of cleaved channels, we compared the proportion of the truncated Ca_v_1.2 form (band of 210 kD) vs. the total amount of Ca_v_1.2 channels (the sum of 210 and 240 kD bands): we observed no differences in whole hippocampal lysates between stressed animals and control animals (Figure 5B).

To determine if the CRS protocol induces an increase in CCAT mRNA in the hippocampus, we measured CCAT transcript levels; as expected from the increase in Ca_v_1.2 transcript levels (Figure 3A), CCAT levels were also increased in whole hippocampus lysates from stressed animals (not shown). However, when CCAT transcript levels were normalized to Ca_v_1.2 transcript levels, no differences were found, indicating that the CRS protocol does not induce promoter activation at exon 46 and that the increased level of CCAT transcripts corresponds to the increased Ca_v_1.2 mRNA levels previously observed (Figure 5C).

Finally, to evaluate CCAT transcriptional activity, we tested if Cx31.1 mRNA level -a gene known to be regulated by CCAT in neurons [27], was different in the hippocampus of stressed animals compared with controls, finding no differences (Figure 5D). Together, these results indicate that the expression or processing of CCAT in the hippocampus is not induced by the CRS protocol.

### 3.4. Changes in CaM Pathways after CRS

**Figure 6:**
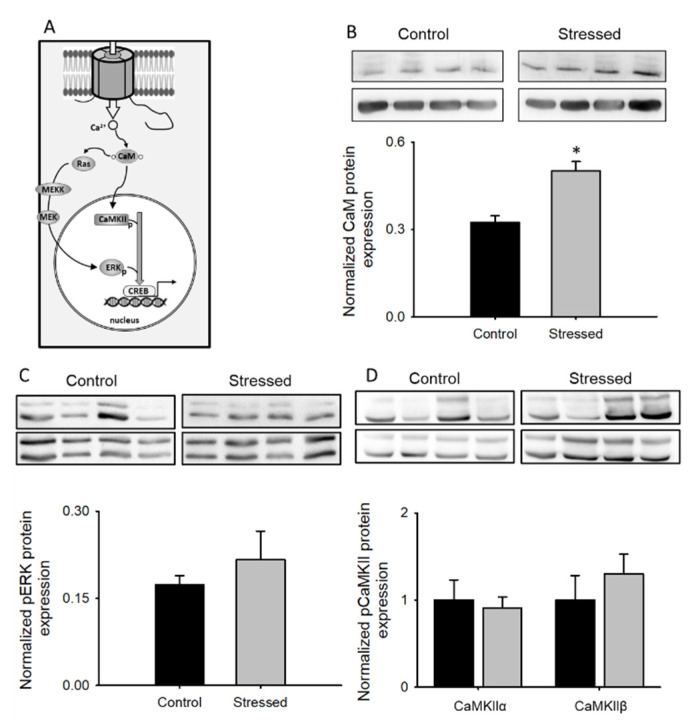
CaM-dependent signaling in CRS-subjected animals: (**A**) Schematic diagram depicting the signaling pathway studied in this figure. (**B**) Bar graph of CaM protein expression from whole hippocampus lysates normalized to α-tubulin (*n* = 8), in the upper part, a representative immunoblot showing CaM and α-tubulin protein expression is shown. Bar graph of pERK (**C**, *n* = 8), pCaMKII (**D**, *n* = 8) expression from whole hippocampus lysates normalized to the total respective protein. Representative Western blots for pERK, pCaMKII, as well as total ERK and CaMKII are shown in the upper part of each bar graph. Bar graphs are mean ± SEM; black bars are control animals, and gray bars correspond to CRS-subjected animals * *p* value < 0.05.

A second main pathway, activated after the opening of the l-type channel, is dependent on calmodulin (CaM). This calcium-binding protein is normally tethered to the Ca_v_1.2 C-terminal at the IQ domain and is involved in two important processes that regulate l-type channel activity: calcium-dependent inactivation (CDI) and calcium-dependent facilitation (CDF) [35,38]. CaM is also involved in the activation of at least three kinases that regulate gene transcription: ERK, CaMKII, and CaMKIV (Figure 6A and Figure S1) [39].

Thus, we next sought to measure CaM levels in the hippocampus of animals subjected to CRS and compare them with control animals. CaM expression in the stressed group was increased by almost 40% compared to the control group (Figure 6B). Activation of the different kinases known to be activated by CaM was studied by determining their phosphorylation levels and normalizing this signal with the total kinase levels.

As seen in Figure 6, no changes were found in the relative phosphorylation levels of ERK (Figure 6C), CaMKII (Figure 6D) or CaMKIV (Figure S1) when total hippocampus lysates from stressed animals were compared with control animals, suggesting that these pathways are unaltered in rats subjected to CRS.

### 3.5. Changes in NFAT Activity after CRS

**Figure 7:**
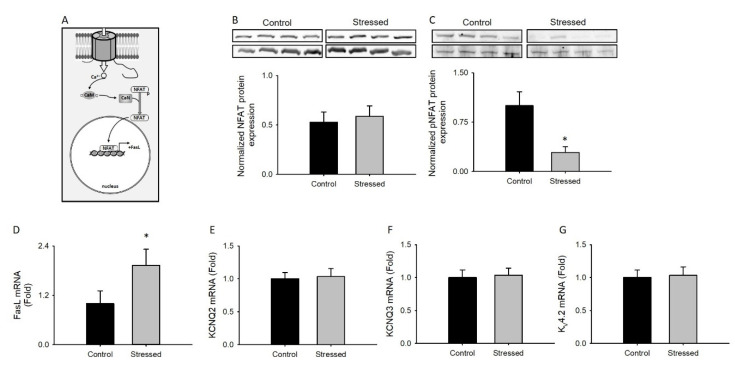
NFAT-dependent signaling in CRS-subjected animals: (**A**) Schematic diagram depicting the signaling pathway studied in this figure. Bar graph of NFAT protein expression normalized to α-tubulin (**B**, *n* = 8) or pNFAT normalized to the total NFAT protein (**C**, *n* = 8) from whole hippocampus lysates; in the upper part of each graph, a representative immunoblot is shown. Bar graph of semi-quantitative PCR expression of genes known to be controlled by NFAT such as FasL mRNA (**D**), KCNQ2 mRNA (**E**), KCNQ3 mRNA (F), or Kv4.2 mRNA (**G**) isolated from whole hippocampus of juvenile rat controls (*n* = 8) or subjected to CRS for 3 weeks (*n* = 8). Bar graphs are mean ± SEM; black bars are control animals, and gray bars correspond to CRS-subjected animals * *p* value < 0.05.

Calcium influx through Ca_v_1.2 channels activates many intracellular pathways that regulate gene transcription, among them, the activation of calcineurin, which dephosphorylates NFAT and promotes its nuclear translocation (Figure 7A). Therefore, we next tested if the CRS protocol induces changes in NFAT expression, phosphorylation, or activity. 

No changes were observed in the total NFAT protein levels in the hippocampus of stressed animals compared to control animals (Figure 7B). In contrast, decreased NFAT phosphorylation levels were observed in the hippocampus of stressed animals (Figure 7C), suggesting increased transcription factor activity.

To examine possible downstream alterations in gene expression as a result of reduced NFAT phosphorylation, and given the observation that the CRS protocol induces neuronal death in the hippocampus [40], we analyzed FasL levels, a well-known NFAT-regulated pro-apoptotic gene. For this, total mRNA was isolated from the hippocampus of stressed and control animals, and FasL transcript levels were measured and normalized with 18S transcript levels. FasL transcripts levels from stressed animals increased by almost two times compared to control animals (Figure 7D), suggesting that the CRS protocol induces apoptosis in the hippocampus. 

Finally, to establish if the observed changes in NFAT activity were specific, we measured the expression of three potassium channels for which transcription is known to be regulated by this transcription factor [41]. The results indicate that there were no differences (Figure 7E–G) and suggest that even though the CRS protocol promotes NFAT dephosphorylation, this pathway is activated in a regulated manner.

## 4. Discussion

In the present study, we demonstrate that male rats subjected to a CRS protocol for 21 days present increased l-type currents in CA1 hippocampal neurons, coherently, we showed an increase in mRNA transcripts and protein levels of the Ca_v_1.2 channel (Figure 3). These results are similar to those from a previous study reporting an increase in Ca_v_1.2 mRNA in the hippocampus following chronic restraint stress in rats [42], but are in contrast with a report that shows no changes in mRNA expression levels after exposing mice to chronic unpredictable stress [13]: this highlights the relevance of the species and the model used.

As confirmed by our own and previous studies, the gain in Ca_v_1.2 function (Figure 4) is related to depression-related behaviors (Figure 1 and Figure 2). This theory arises from many studies, including the initial observation showing that nifedipine, a well-known l-type calcium channel blocker, has an antidepressant-like effect in rodents [43], while the enhancement of l-type calcium currents with BayK8644 induces a depressive-like phenotype [44]. In accordance, partial genetic ablation of the CACNA1C gene (Ca_v_1.2 KO heterozygous mice) exhibited an antidepressant-like behavior [14]. CACNA1C polymorphisms associated with depression have also been linked to increased Ca_v_1.2 mRNA and l-type calcium currents in induced neurons obtained from skin fibroblasts of patients with depressive-like behaviors [12]. 

In neurons, Ca_v_1.2 channels are critical mediators of Ca^2+^ signaling [45]. Ca^2+^ influx through these channels activates the calcium sensor calmodulin (CaM), which may subsequently activate a series of protein kinases such as CaMK (CaMKII and CaMKIV) and MAPK, both of which transduce molecular cascades to the nucleus [39]. An increase in their kinase activity is related to LTP consolidation and antidepressant effects [46], thus it is not surprising that we did not find changes in phosphorylation levels of these kinases associated with CaM (Figure 6 and supplementary Figure S1). Nevertheless, we did find an increase in CaM expression in the hippocampus of stressed animals (Figure 6), suggesting that this pathway is augmented in depression.

Apart from kinase activation, increased CaM activity may lead to the activation of phosphatase pathways, modulating, for example, the function of the nuclear factor of activated T cells (NFAT) family of transcription factors [26]. NFAT4c phosphorylation levels are decreased in the hippocampus of animals subjected to CRS (Figure 7), suggesting that this transcription factor translocate from the cytoplasm to the nucleus. The activation of l-type calcium channels by high extracellular K^+^, *N*-methyl-d-aspartate treatment and brain-derived neurotrophic factor application has been shown to induce NFATc4 nuclear translocation in hippocampal neurons [47,48], a process normally associated with the activation of calcineurin. As this phosphatase is a well-known calcium/calmodulin-regulated protein phosphatase, is likely to be responsible for the observed NFAT4c dephosphorylation. Interestingly, calcineurin-dependent NFAT4c translocation has been recently involved in many neurological disorders such as Alzheimer’s disease [49], traumatic brain injury [50], postoperative cognitive dysfunction [51], and post-traumatic stress (PTSD) [52].

Among the various genes that are regulated by NFAT4c, here we showed increased FasL mRNA levels (Figure 7): we propose that this could be the molecular mechanism accounting for the observed hippocampal apoptosis induced by depression [40,53,54]. In fact, apoptotic markers at the hippocampus have been detected in several animal models for mood disorders like PTSD [55], predatory stress [56], social defeat [57], and unpredictable stress [58], but the initiation signaling is lesser-known.

Several cerebral regions are central to the understanding of the pathophysiology of mood disorders. Thus, the effect of chronic stress protocols on different brain areas could vary at the cellular level. In this sense, significant knowledge has been obtained regarding the role of Ca_v_1.2 at the prefrontal cortex (PFC). Induced local knockdown of CaCNA1C in the adult PFC is sufficient to induce anxiety-like behavior [14], and, in this animal model, PFC neurons exhibit lower depression-related protein (REDD1) expression levels, and restoring these levels is enough to reverse the antidepressant-like phenotype [13,14]. 

In contrast, the Ca_v_1.2 gain of function effect in the hippocampus is largely unknown [59]. There is a significant reduction in hippocampal volume in patients with depression, with some studies indicating that this hippocampal volumetric change is caused by the apoptosis of hippocampal neurons [40,53] (however, see [60]). The data presented here shed light on this topic, in the sense that they are coherent with a model where increased Ca_v_1.2 activity (Figure 4) induced activation of the CaM-calcineurin-NFATc4 axis (Figure 6 and Figure 7) leading to FasL-dependent neuronal apoptosis at the hippocampus. However, further experiments are needed to corroborate this idea.

## Figures and Tables

**Table 1:** List of primers.

Target	Sense	Antisense
18S	gggcccgaacgctttacttt	ttgcgccggtccaagaattt
CCAT	tcccaagttcatcgaggtca	aaggtaagagggtgccgttg
Ca_v_1.2	ccgctttgactgtttcattgtg	cgaggttgctcagggagttc
Cx31.1	ctcgagcccacgtgaagaaa	actccgactcagctcttttcc
FasL	atccctctggaatgggaaga	ccatatctggccagtagtgc
KNNQ2	gcgttcctttagcggtttca	aagactgcggattgcatcct
KCNQ3	gcaaatgccatgccttgaga	aagactgcggattgcatcct
K_v_4.2	tgcttcactgcctggtttca	ttcttgcacgctgcctctat

**Table 2:** List of antibodies.

Antibody	Type	Dilution	Source	Catalog Number
Calmodulin	pAB	1:200	ABCAM	ab155550
CaMKII	pAB	1:1000	Cell Signaling	44365
CaMKIV	mAB	1:100	Santa Cruz	55501
Ca_v_1.2	pAB	1:200	Alomone	ACC-003
ERK	mAB	1:1000	Cell Signaling	9102
NFAT4c	pAB	1:100	Santa Cruz	SC-13036
pCaMKII-T286	mAB	1:1000	Cell Signaling	127165
pCaMKIV	pAB	1:1000	ABCAM	ab59424
pERK	mAB	1:1000	Cell Signaling	9101
pNFAT4c	mAB	1:2000	Santa Cruz	135770
Tubulin	pAB	1:3000	Santa Cruz	SC-20172

**Table 3:** Sample-size per test.

Test	Sample Size	
	Control	CRS *
Body weight determination	24	30
Adrenal gland determination	9	9
Forced swim test	19	26
Sucrose preference test	24	30
Social interaction test	15	15
Electrophysiology	7	7
Western Blot	12	12
Semi-quantitative PCR	12	12

* Chronic Restraint Stress (CRS).

## Supplementary Materials

The following are available online at https://www.mdpi.com/2073-4409/9/12/2609/s1, Figure S1: CaMIV phosphorylation level in CRS-subjected animals.
