# Supplementary material for: Cav1.2 Activity and Downstream Signaling Pathways in the Hippocampus of An Animal Model of Depression
Source: Cells. 2020 Dec 4;9(12):2609. doi: 10.3390/cells9122609 (PMC7762021; doi:10.3390/cells9122609)
Supplement: Supplementary file 1 [file cells-09-02609-s001.pdf]

Supplementary Figure 1

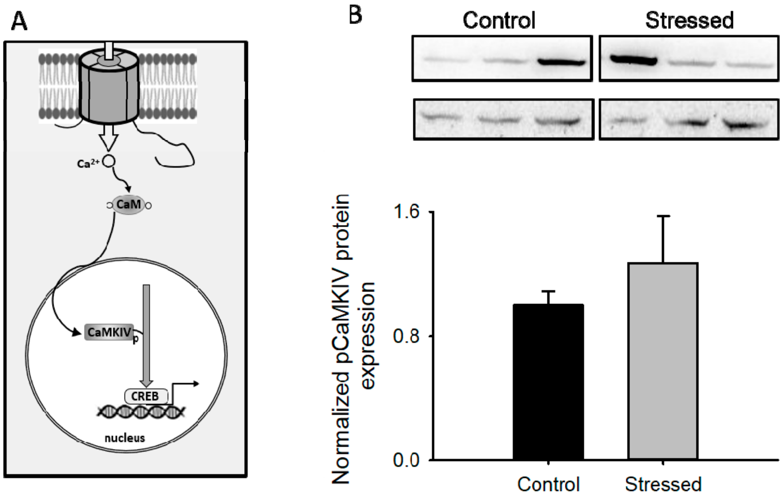

Supplementary Figure 1. CaMIV phosphorylation level in CRS-subjected animals: *(A) Schematic diagram depicting the signalling pathway studied in this figure. (B) Bar graph of pCaMKIV (n=4) expression from whole hippocampus lysates normalized to the total respectively protein. Representative western blots for pCaMKIV, and total CaMKIV is shown in the upper part of the bar graph. Bar graphs are mean ± SEM; black bars are control animals, and gray bars correspond to CRS-subjected animals. Given the reduced sample-size, a power of 80% was used in this statistical analysis.*
